# Calorie labeling and consumer estimation of calories purchased

**DOI:** 10.1186/s12966-014-0091-2

**Published:** 2014-07-12

**Authors:** Glen B Taksler, Brian Elbel

**Affiliations:** 1 Medicine Institute, Cleveland Clinic, 9500 Euclid Avenue, G1-40F, Cleveland 44195, OH, USA; 2 Departments of Population Health and Medicine, New York University School of Medicine, New York, NY, USA; 3 New York University Wagner School of Public Service, New York, NY, USA

**Keywords:** Diet, Health policy, Energy intake, Caloric restriction, Obesity

## Abstract

**Background:**

Studies rarely find fewer calories purchased following calorie labeling implementation. However, few studies consider whether estimates of the number of calories purchased improved following calorie labeling legislation.

**Findings:**

Researchers surveyed customers and collected purchase receipts at fast food restaurants in the United States cities of Philadelphia (which implemented calorie labeling policies) and Baltimore (a matched comparison city) in December 2009 (pre-implementation) and June 2010 (post-implementation). A difference-in-difference design was used to examine the difference between estimated and actual calories purchased, and the odds of underestimating calories.

Participants in both cities, both pre- and post-calorie labeling, tended to underestimate calories purchased, by an average 216–409 calories. Adjusted difference-in-differences in estimated-actual calories were significant for individuals who ordered small meals and those with some college education (accuracy in Philadelphia improved by 78 and 231 calories, respectively, relative to Baltimore, p = 0.03-0.04). However, categorical accuracy was similar; the adjusted odds ratio [AOR] for underestimation by >100 calories was 0.90 (p = 0.48) in difference-in-difference models. Accuracy was most improved for subjects with a BA or higher education (AOR = 0.25, p < 0.001) and for individuals ordering small meals (AOR = 0.54, p = 0.001). Accuracy worsened for females (AOR = 1.38, p < 0.001) and for individuals ordering large meals (AOR = 1.27, p = 0.028).

**Conclusions:**

We concluded that the odds of underestimating calories varied by subgroup, suggesting that at some level, consumers may incorporate labeling information.

## 

Calorie labeling legislation has been introduced in several United States cities and states to reduce obesity rates. Nationally, the Patient Protection and Affordable Care Act is expected to require restaurants with ≥20 locations to post calories for all regular food and drink items [[1]].

Yet, studies suggest that calorie labeling has little impact on the number of calories purchased. Studies from Philadelphia [[2]] and low-income areas in New York City [[3]] found that labeling was associated with consumers noticing calorie labels but no significant change in calories purchased. Most other controlled studies have found similar results [[4]-[7]], although one study found that consumers at Starbucks purchased 12 fewer calories following calorie labeling [[8]]. Experimental studies have found mixed results [[9],[10]].

Despite little evidence of a change in number of calories purchased, recent work has considered whether labeling is associated with greater accuracy in estimates of the number of calories purchased [[11]]. That is, while consumers purchase a similar number of calories, do they better judge the caloric content of foods following labeling policies? Such a finding could indicate that, at some level, consumers absorb calorie labeling information. Given the time associated with behavior change, such a mechanism could indicate an important first step in the potential longer-term impact of labeling. One prior study suggests that consumers were 9 percentage points more accurate in correctly predicting calories purchased (within 100 calories, from 15% before labeling to 24% after labeling) [[11]], but was limited to New York City. Other prior work has attributed caloric underestimation to a lack of visual cues [[12],[13]]. In one study, subjects who ate from self-refilling soup bowls (lacking the visual control of a bowl for portion size) were found to consume 73% more soup than controls; however, both groups estimated similar caloric consumption [[12]]. Caloric underestimation may also be related to nutritional status (overestimation of energy content for unhealthy foods) [[14]], less overall health consciousness [[15]], and lower education [[16]]. More generally, food labels appear most often used when easier-to-understand [[17],[18]], though some literature suggests an association to health literacy [[19]-[22]], female gender [[21]-[23]], and higher education [[21],[22]].

Using a larger and more diverse sample than previous research, researchers examine the influence of calorie labeling on estimation of calories purchased in Philadelphia.

## Findings

### Methods

Data were collected as part of a larger study to examine the influence of calorie labeling implemented in Philadelphia in 2010 [[2]]. A difference-in-difference design was used to examine the difference between estimated and actual calories purchased in Philadelphia in December 2009 (pre-calorie labeling) versus June 2010 (post-calorie labeling), as compared to Baltimore (a matched comparison city without calorie labeling rules) during the same month. The Appendix describes difference-in-difference methodology in more detail. Baltimore was selected as the city most comparable to Philadelphia by calculating Euclidean distances between Philadelphia and each of the largest 100 US cities using standardized city-level measures derived from Census 2000 data, including population size, poverty, unemployment, education, race/ethnicity, and income measures [[2]]. Full methods are available elsewhere [[2]].

Research staff stood outside locations of McDonald’s and Burger King during lunch (approximately 11:30 am-2:30 pm) or dinner (approximately 5:00 pm-8:00 pm) on weekdays, and approached entering customers appearing to be ≥18 years old and asked them to bring back their receipt in exchange for $2 [[2]]. Participants who agreed were asked questions including which items were ordered for him/herself (versus other individuals); the exact nature of items (added cheese, mayonnaise, etc.); how often they visited “big chain” fast food restaurants; and how many calories they estimated to be in their purchase. The receipt provided was used to calculate actual calories purchased, based on nutrition information provided by each restaurant (as of May 2010) [[2]].

First, summary statistics were calculated for the full sample (N = 1835) and subgroups based on number of calories purchased (≤median [850 calories] vs. >median), gender, race/ethnicity, education, and food vs. beverage. Summary statistics were calculated for each city, both pre- and post-calorie labeling. T-tests of unadjusted statistical significance were run for 4 groups: Philadelphia vs. Baltimore pre-calorie labeling, Philadelphia vs. Baltimore post-calorie labeling, Philadelphia pre- versus post-calorie labeling, and Baltimore pre- versus post-calorie labeling.

Researchers then examined the difference between estimated and actual calories using multiple regression models. The dependent variable was estimated minus actual calories for each respondent. A positive number meant an overestimate and a negative number meant an underestimate of actual calories. The key independent variable of interest was an interaction term between Philadelphia (versus Baltimore) and post-calorie labeling (versus pre-calorie labeling). That is, researchers sought to measure the marginal contribution of calorie labeling policies to the accuracy of estimates in Philadelphia. Independent covariates included age, gender, race/ethnicity, education, number of items purchased, purchase of a combination meal, to-go vs. eat-in consumption, number of fast food restaurant visits per week, city, and time period (pre- vs. post-calorie labeling).

Finally, consistent with prior research suggesting that consumers tend to underestimate calories [[2],[3],[11],[24]], logistic regression models were used to consider whether subjects underestimated by >100, >250, and >500 calories. (Researchers verified that consumers in the sample, on average, underestimated calories; results shown below.) This analysis was used to consider broad patterns in accuracy pre- vs. post-calorie labeling, as opposed to the magnitude difference between estimated and actual calories. Odds ratios were adjusted for the same covariates described above.

Standard errors were clustered by restaurant. Tests were performed with a two-sided alpha = 0.05. This study was approved by the Institutional Review Board of New York University School of Medicine.

## Results

Table 1 presents summary statistics. Respondents were primarily male, black or African American, and held a high school or lower education. No significant differences were observed in the actual number of calories purchased, though some differences existed across cities (a larger proportion of females in Philadelphia, and larger proportion of blacks and fast food visits/week in Baltimore) and time periods (a larger proportion of females and blacks in Philadelphia, and less missing data in Baltimore, in the post-calorie labeling period).

**Table 1:** Summary statistics

	**All**		**Philadelphia**				**Baltimore**				**Significance tests**			
			**Pre-**		**Post-**		**Pre-**		**Post-**		**Pre-**	**Post-**	**Pre vs. Post**	
	**Mean**	**SD**	**Mean**	**SD**	**Mean**	**SD**	**Mean**	**SD**	**Mean**	**SD**			**Philadelphia**	**Baltimore**
N	1835		470		534		394		437					
***Mean***														
Age	39.1	13.9	39.7	14.1	37.4	14.4	40.8	13.5	38.9	13.4			*	*
Number of calories purchased, actual	951	685	987	757	927	704	974	696	923	559				
***Percent***														
Gender														
Male	55.2	49.8	58.3	49.4	52.1	50.0	51.5	50.0	58.8	49.3	*	*	*	
Female	37.4	48.4	37.5	48.5	45.5	49.8	28.9	45.4	35.2	47.8	**	**	**	
Missing	7.4	26.2	4.3	20.2	2.4	15.4	19.5	39.7	6.0	23.7	***	**		***
Race/Ethnicity														
Black	70.1	45.8	60.4	49.0	70.8	45.5	73.9	44.0	76.4	42.5	***	*	***	
Caucasian	20.8	40.6	23.0	42.1	17.8	38.3	22.1	41.5	21.1	40.8			*	
Other/Missing	4.1	19.8	6.0	23.7	5.2	22.3	3.3	17.9	1.4	11.7		**		
Education														
High school or less	60.9	48.8	54.7	49.8	63.3	48.2	62.2	48.6	63.4	48.2	*		**	
Some college or AA	25.2	43.4	29.8	45.8	23.0	42.1	22.6	41.9	25.2	43.5	*		*	
BA or above	10.6	30.8	11.7	32.2	8.1	27.2	12.4	33.0	10.8	31.0				
Missing	3.4	18.1	3.8	19.2	5.6	23.1	2.8	16.5	0.7	8.3		***		*
Type of order														
To go	67.6	46.8	60.6	48.9	70.4	45.7	68.0	46.7	71.4	45.2	*		**	
Eat in	26.3	44.1	25.1	43.4	24.2	42.8	28.4	45.2	28.4	45.1				
Missing	6.1	23.9	14.3	35.0	5.4	22.7	3.6	18.5	0.2	4.8	***	***	***	***
Number of times usually eat in big chain fast food restaurant per week														
≤1	56.4	49.6	62.3	48.5	64.0	48.0	49.0	50.1	47.1	50.0	***	***		
2	15.8	36.4	12.3	32.9	12.7	33.4	17.5	38.1	21.5	41.1	*	***		
≥3	34.4	47.5	26.4	44.1	30.7	46.2	39.6	49.0	42.8	49.5	***	***		
Missing	3.1	17.4	7.7	26.6	2.4	15.4	1.5	12.3	0.5	6.8	***	*	***	
Number of items purchased														
1	23.2	42.2	18.5	38.9	25.7	43.7	25.9	43.9	22.7	41.9	**		**	
2	20.0	40.0	21.5	41.1	18.9	39.2	19.0	39.3	20.4	40.3				
3	31.6	46.5	31.7	46.6	32.2	46.8	31.0	46.3	31.4	46.4				
4	11.3	31.7	10.2	30.3	9.2	28.9	12.2	32.8	14.4	35.2		*		
≥5	14.0	34.7	18.1	38.5	14.0	34.8	11.9	32.5	11.2	31.6	*			
Purchased combination meal	24.5	43.0	21.5	41.1	25.7	43.7	25.4	43.6	25.6	43.7				
Restaurant														
McDonald’s	64.2	48.0	66.2	47.4	70.2	45.8	61.7	48.7	57.0	49.6		***		
Burger King	35.8	48.0	33.8	47.4	29.8	45.8	38.3	48.7	43.0	49.6		***		

***P < 0.001, **P < 0.01, *P < 0.05.

Table 2 shows regression results for the difference between estimated and actual calories. In the full sample and every subgroup, participants in both cities and time periods tended to underestimate calories purchased, by an average of 216–409 calories. The difference-in-difference coefficient was typically positive, meaning that respondents in Philadelphia were more accurate relative to Baltimore post-calorie labeling, but was only significant for 2 subgroups: respondents who purchased ≤ median number of calories (coefficient = 78, p = 0.04) and respondents with some college education (coefficient = 231, p = 0.03).

**Table 2:** Actual versus estimated calories, Philadelphia versus Baltimore

	**Actual**		**Estimated**		**Estimated minus actual**				**Difference-in-Difference**		
	**Pre**	**Post**	**Pre**	**Post**	**Pre**	**Post**	**Significance tests**		**Unadj**	**Adj (95% CI)**	**P**
							**Pre**	**Post**			
**Full sample**											
Philadelphia	987	927	578	581	−409	−346	**		177	122 (−809, 1052)	0.35
Baltimore	974	923	758	593	−216	−330					
**Purchased >850 calories (median)**											
Philadelphia	1480	1450	780	758	−700	−692	*		223	191(−2301,2682)	0.51
Baltimore	1430	1390	1032	777	−398	−613					
**Purchased ≤850 calories (median)**											
Philadelphia	446	459	357	422	−89	−37			105	78 (20, 136)	0.04
Baltimore	463	486	450	420	−13	−65					
**Male**^**1**^											
Philadelphia	982	943	575	609	−407	−334			130	124 (−998, 1245)	0.39
Baltimore	1006	968	692	597	−314	−370					
**Female**^**1**^											
Philadelphia	987	925	602	562	−385	−363			−41	−87 (−386, 213)	0.17
Baltimore	993	834	689	591	−305	−243					
**Black**^**1**^											
Philadelphia	933	858	543	585	−389	−273			173	100 (−760, 959)	0.38
Baltimore	1007	895	745	577	−262	−318					
**White**^**1**^											
Philadelphia	1088	990	684	751	−405	−239			384	250 (−524, 1025)	0.15
Baltimore	886	950	815	661	−71	−290					
**High school or less**^**1**^											
Philadelphia	968	885	545	475	−423	−409			169	54 (−590, 698)	0.48
Baltimore	954	934	698	523	−256	−411					
**Some college or AA**^**1**^											
Philadelphia	1028	977	582	811	−447	−166			170	231 (77, 385)	0.03
Baltimore	1065	914	758	718	−307	−196	*				
**BA or above**^**1**^											
Philadelphia	1065	1141	650	696	−414	−445	*		149	231(−2138,2600)	0.43
Baltimore	968	900	919	671	−49	−229					
**Food only**											
Philadelphia	801	691	521	528	−279	−163			180	205 (−514, 924)	0.17
Baltimore	774	719	618	500	−156	−219					
**Beverage only**											
Philadelphia	203	308	204	231	1	−77			−13	−60 (−1450,1329)	0.68
Baltimore	306	368	341	338	35	−31					
**Purchased 1 item**											
Philadelphia	320	316	221	286	−99	−30	**		167	181 (−864, 1226)	0.27
Baltimore	319	339	364	286	45	−53					
**Purchased >1 item**											
Philadelphia	1139	1138	660	683	−480	−455	*		128	112 (−932, 1156)	0.40
Baltimore	1202	1093	895	683	−307	−411					
**Purchased combination meal**											
Philadelphia	1441	1512	768	738	−674	−774			9	−15(−2050,2019)	0.94
Baltimore	1482	1383	932	723	−550	−659					
**Did not purchase combination meal**											
Philadelphia	863	725	527	527	−337	−198	**		252	167 (−539, 872)	0.20
Baltimore	801	764	698	548	−102	−216	*				

^1^May not sum to the full sample because of missing gender, race, and/or education for some subjects.

Unadj: Unadjusted. Adj: Adjusted.

**P < 0.01, *P < 0.05.

Table 3 shows the logistic regression results for subjects’ likelihood to underestimate calories, versus overestimating or correctly estimating calories. In the full sample, the odds of underestimation by >100 calories was similar post- vs. pre-calorie labeling legislation, with an adjusted odds ratio[AOR] of 0.90 (95% = 0.67-1.21, p = 0.48). However, gross underestimates were less likely; the AOR for underestimation by >500 calories was 0.75 (95% CI = 0.73-0.77, p < 0.001). Accuracy in Philadelphia post-calorie labeling was most improved for subjects with a BA or higher education (AOR = 0.25, 95% CI = 0.12-0.50, p < 0.001) and for subjects ordering less than the median number of calories (AOR = 0.54, 95% CI = 0.37-0.78, p = 0.001). Accuracy deteriorated among females (AOR = 1.38, p < 0.001), respondents who purchased more than the median number of calories (AOR = 1.27, p = 0.028), and respondents who purchased a combination meal (AOR = 1.23, p = 0.012).

**Table 3:** Error in estimate of number of calories purchased, Philadelphia vs. Baltimore

	**Philadelphia**		**Baltimore**		**Difference-in-Difference**		
	**Pre-**	**Post-**	**Pre-**	**Post-**	**Unadj.**	**Odds ratio**	**P**
						**(95% CI)**	
*Percent*							
*Error in estimate of number of calories (kcal) purchased*							
**Full sample, correct within 100 kcal**							
Overestimated by >100 kcal	11.9	14.4	25.6	15.3			
Correctly estimated within 100 kcal	18.9	15.5	10.2	14.2			
Underestimated by >100 kcal	69.2	70.0	64.2	70.5	−5.4	0.90 (0.67-1.21)	0.48
**Full sample, correct within 250 kcal**							
Overestimated by >250 kcal	9.6	10.3	21.6	11.7			
Correctly estimated within 250 kcal	32.6	34.8	29.7	33.9			
Underestimated by >250 kcal	57.9	54.9	48.7	54.5	−8.7	0.82 (0.65-1.04)	0.095
**Full sample, correct within 500 kcal**							
Overestimated by >500 kcal	6.8	5.6	14.5	7.3			
Correctly estimated within 500 kcal	53.6	58.8	53.3	55.6			
Underestimated >500 kcal	39.6	35.6	32.2	37.1	−8.8	0.75 (0.73-0.77)	<0.001***
**Purchased >850 kcal (median)**							
Overestimated by >100 kcal	9.8	11.1	25.0	8.5			
Correctly estimated within 100 kcal	7.3	4.8	3.4	6.2			
Underestimated >100 kcal	82.9	84.1	71.6	85.3	−12.5	1.27 (1.03-1.56)	0.028*
**Purchased ≤850 kcal (median)**							
Overestimated by >100 kcal	14.3	17.4	26.3	21.7			
Correctly estimated within 100 kcal	31.7	25.2	17.7	21.7			
Underestimated >100 kcal	54.0	57.5	55.9	56.6	2.7	0.54 (0.37-0.78)	0.001**
**Male**							
Overestimated by >100 kcal	12.0	16.2	20.7	14.0			
Correctly estimated within 100 kcal	17.5	16.6	9.9	14.4			
Underestimated >100 kcal	70.4	67.3	69.5	71.6	−5.3	0.81 (0.60-1.08)	0.15
**Female**							
Overestimated by >100 kcal	11.4	13.2	21.9	17.5			
Correctly estimated within 100 kcal	20.5	13.6	8.8	14.3			
Underestimated >100 kcal	68.2	73.3	69.3	68.2	6.2	1.38 (1.25-1.53)	<0.001***
**Black**							
Overestimated by >100 kcal	12.0	15.3	25.4	15.0			
Correctly estimated within 100 kcal	22.2	16.1	8.6	13.5			
Underestimated >100 kcal	65.9	68.5	66.0	71.6	−2.9	0.96 (0.60-1.52)	0.86
**White**							
Overestimated by >100 kcal	13.9	14.7	26.4	17.4			
Correctly estimated within 100 kcal	14.8	10.5	13.8	16.3			
Underestimated >100 kcal	71.3	74.7	59.8	66.3	−3.1	1.29 (0.85-1.96)	0.22
**High school or less**							
Overestimated by >100 kcal	8.6	12.1	26.5	11.9			
Correctly estimated within 100 kcal	21.4	14.8	9.0	13.0			
Underestimated >100 kcal	70.0	73.1	64.5	75.1	−7.6	0.82 (0.60-1.13)	0.22
**Some college or AA**							
Overestimated by >100 kcal	13.6	18.7	21.4	20.0			
Correctly estimated within 100 kcal	15.7	17.1	7.9	16.4			
Underestimated >100 kcal	70.7	64.2	70.8	63.6	0.7	1.16 (0.93-1.44)	0.18
**BA or above**							
Overestimated by >100 kcal	14.6	20.9	26.5	23.4			
Correctly estimated within 100 kcal	12.7	18.6	16.3	14.9			
Underestimated >100 kcal	72.7	60.5	57.1	61.7	−16.8	0.25 (0.12-0.50)	<0.001***
**Food only**							
Overestimated by >100 kcal	10.4	16.7	25.2	15.3			
Correctly estimated within 100 kcal	19.8	16.1	10.3	19.6			
Underestimated >100 kcal	69.8	67.3	64.5	65.0	−3.0	0.88 (0.44-1.81)	0.77
**Beverage only**							
Overestimated by >100 kcal	14.6	20.9	26.5	23.4			
Correctly estimated within 100 kcal	12.7	18.6	16.3	14.9			
Underestimated >100 kcal	72.7	60.5	57.1	61.7	−16.8	1.71 (0.12-12.76)	0.63
**Purchased 1 item**							
Overestimated by >100 kcal	12.6	17.5	27.5	18.2			
Correctly estimated within 100 kcal	40.2	32.9	24.5	24.2			
Underestimated >100 kcal	47.1	49.6	48.0	57.6	−7.0	0.73 (0.34-1.60)	0.44
**Purchased >1 item**							
Overestimated by >100 kcal	11.8	13.4	25.0	14.5			
Correctly estimated within 100 kcal	14.1	9.6	5.1	11.2			
Underestimated >100 kcal	74.2	77.1	69.9	74.3	−1.5	0.97 (0.81-1.15)	0.72
**Purchased combination meal**							
Overestimated by >100 kcal	15.8	8.8	21.0	8.9			
Correctly estimated within 100 kcal	6.9	5.1	2.0	5.4			
Underestimated >100 kcal	77.2	86.1	77.0	85.7	0.2	1.23 (1.05-1.44)	0.012*
**Did not purchase combination meal**							
Overestimated by >100 kcal	10.8	16.4	27.2	17.5			
Correctly estimated within 100 kcal	22.2	19.1	12.9	17.2			
Underestimated >100 kcal	66.9	64.5	59.9	65.2	−7.8	0.84 (0.61-1.16)	0.29

Unadj.,Unadjusted. kcal: Calories.

***P < 0.001, **P < 0.01, *P < 0.05.

## Discussion

Numerous studies suggest that respondents purchase a similar number of calories pre- and post-calorie labeling [[3]-[5]]. This result has often been interpreted as suggesting that consumers do not use calorie-labeling information.

Researchers found that consumers in Philadelphia, which implemented calorie-labeling policies, were less likely to grossly underestimate calories (by >500 calories) post-labeling, relative to Baltimore, which did not implement such policies. These results suggest that at some level, consumers may incorporate labeling information, a novel result. Categorical accuracy for underestimation by >100 calories varied widely by subgroup, with improved accuracy among more educated consumers and those ordering small meals, and lower accuracy among women, consumers ordering large meals, and consumers ordering combination meals. No significant differences by race were found. Further research exploring why consumers choose to purchase a high number of calories despite increased awareness of the number of calories purchased is needed.

Perhaps most notably, respondents with a BA education or higher had a 75% reduction in odds for underestimating by >100 calories in Philadelphia post- versus pre-labeling (Table 3). This finding suggests that public health campaigns to promote understanding of calorie labeling may best be centered around less educated populations, who are less likely to report using posted information [[2]]. While females had 38% increased odds for underestimating by >100 calories post-calorie labeling (Table 3), this finding may be tempered by an 8.1 percentage point increase in the proportion of females in Philadelphia post-calorie labeling (p = 0.010, Table 1), compared with an insignificant change in the proportion of females in Baltimore (p = 0.053, Table 1). We therefore would be cautious not to overinterpret differences in use of calorie labeling by gender, although some prior work in psychology has found greater calorie underestimation by women [[25]]. Additionally, while consumers could have purchased differently as a result of the survey or incentive ($2), the data collection procedures were consistent across all periods and locations, suggesting that this should not influence the impact estimates [[2]].

We also found that the odds of underestimating calories post-calorie labeling declined in respondents who purchased ≤ median number of calories (AOR = 0.54, p < 0.001) but increased in respondents who purchased > median calories (AOR = 1.27, p = 0.028) (Table 3). Since respondents who purchased combination meals bought twice as many calories as other respondents (medians = 1340 and 670 calories, respectively), it is possible that calorie labels for combination meals were more confusing. These calorie labels typically gave wider ranges (“500-2000 calories”) that required individuals wanting further information to look-up calories for each item in the combination meal. Future research should consider whether providing more detailed information on combination meal calorie labels might improve overall accuracy.

## Competing interests

The authors declare that they have no competing interests.

## Authors’ contributions

GBT was involved in conceptualizing the study, design, analysis plan, interpretation of results, and writing. BE was involved in conceptualizing the study, design, interpretation and writing. Both authors read and approved the final manuscript.

## Appendix

The change in calories purchased in Philadelphia post-calorie labeling legislation was assumed to derive from two potential factors, calorie labeling legislation or secular trends. To measure secular trends, researchers surveyed calories purchased in Baltimore, a control city, during the same time periods as for Philadelphia. Researchers assumed that the change in calories purchased in Baltimore would represent the secular trend, and any remaining change in calories purchased would be due to calorie labeling legislation. The difference in calories purchased in Philadelphia, relative to the change in calories purchased in Baltimore, is sometimes called the “difference-in-difference.” The regression model was as follows:

(1) $$y\;=a+\beta_{0}\times \left[\mathrm{Philadelphia}\right]\;+\beta_{1}\times \left[\mathrm{Post}\right]\;+\beta_{2}\times \left[\mathrm{Philadelphia}\;*\;\mathrm{Post}\right]\;+\delta \times \left[X\right]\;+\varepsilon$$

where *α =* constant; *Philadelphia* = 1 if Philadelphia, 0 if Baltimore; *Post* = 1 if post-calorie labeling legislation, 0 if pre-calorie labeling legislation; ***X*** = an array of all other independent variables (with a corresponding array of coefficient estimates *δ)*; and *ε* = error term.

*β*_*2*_*,* the interaction between Philadelphia and post-calorie labeling legislation, represented the difference-in-difference estimate.
